# Optimising breast cancer screening in national mammography screening centres: challenges and insights on implementing additional ultrasound for women with dense breast tissue — a qualitative study

**DOI:** 10.1186/s12885-025-15145-1

**Published:** 2025-10-31

**Authors:** Susanne A. Elsner, Eva Haußmann, Paula Grieger, Moritz Hadwiger, Andrea Rieck, Astrid Hacker, Sylvia Heywang-Köbrunner, Alexander Katalinic

**Affiliations:** 1 Institute for Social Medicine and Epidemiology, University of Lübeck, Lübeck, Germany; 2 Mammography Reference Centre Munich, Munich, Germany

**Keywords:** Breast density, Mammography screening, Ultrasound examination, Diagnostic sensitivity, Patients knowledge, Healthcare provider experiences, Health care intervention, Qualitative study, DIMASOS study

## Abstract

**Background:**

Research indicates that incorporating supplemental breast ultrasound in addition to mammography can enhance the sensitivity of public mammography screening, particularly for women with dense breast tissue. However, little information exists regarding the feasibility of integrating ultrasound into public mammography screening. Moreover, limited data exist on the awareness, perceptions, and experiences of both screening clients and healthcare providers regarding dense breast tissue, its impact on screening effectiveness, and the acceptability and implications of incorporating additional ultrasound.

**Methods:**

In a prospective, controlled mixed-methods study, 31 semi-structured telephone interviews were conducted with both screening professionals and clients identified as having dense breast tissue. Six mammography screening centres in Germany participated in the qualitative part of the DIMASOS2 study (Density-Indicated Mammographic-Sonographic Breast Cancer Screening), where additional ultrasound was offered to women aged 50 to 69 years identified as having dense breast tissue. The interviews were analysed using qualitative content analysis.

**Results:**

Clients expressed appreciation for the additional ultrasound examination; half of them were already aware of their dense breast tissue but lacked knowledge about its implications for diagnostic accuracy. Healthcare professionals felt that diagnostic sensitivity of mammography screening needed to be improved, but also faced challenges such as insufficient evidence for adjunct measures, concerns about overdiagnosis and false positives, and substantial organisational barriers to implementing ultrasound examinations.

**Conclusions:**

This study highlights a gap in open discussions about breast density and its impact on screening accuracy. The absence of evidence-based information hampers informed decision making for both healthcare providers and clients. Moreover, substantial organisational constraints hinder the incorporation of breast ultrasound into public screening programme, leading to a complex and unsatisfactory situation for healthcare professionals and clients.

**Supplementary Information:**

The online version contains supplementary material available at 10.1186/s12885-025-15145-1.

## Background

The public breast cancer screening programme in Germany is aimed at women aged 50–75 years; the upper age limit was raised from 69 to 75 in July 2024. Eligible women are invited to participate in the programme every two years. Some studies suggest that public mammography screening is associated with a relative reduction in breast cancer mortality of approximately 20%, mainly due to earlier diagnosis and intervention [1, 2].

The sensitivity of mammography ranges between 77 and 95%, and specificity between 94 and 97% [3, 4]. However, sensitivity is significantly lower in women with dense and very dense breast tissue, as density can mask cancers on mammograms [5–8]. In European screening programmes, approximately 5–10% of women are affected by very dense breast tissue, which can significantly impair diagnostic accuracy [8–10]. In approximately 30% of women with heterogeneously dense tissue, diagnostic accuracy is also impaired, albeit to a lesser extent. Due to a higher incidence of breast cancer and the masking effect of dense tissue, these women face an increased risk of delayed diagnosis and interval tumours, which often have a worse prognosis than tumours detected through regular screening [7, 11–15].

Imaging techniques such as breast ultrasound (US) have been shown to enhance diagnostic accuracy by improving sensitivity for women with dense breasts when used in addition to mammography [16–18]. US appears to be a safe and relatively cost-effective supplemental method in addition to mammography. A recent Cochrane review reported an additional cancer detection rate of 2–3 cases per 1 000 examinations when US was combined with mammography in women with dense breasts [3].

In Germany, as in many other countries, there is currently no expert consensus or formal guideline on supplemental screening for women with dense breast tissue who have a negative mammogram. Existing guidelines discouraging additional screening refer to the potential harms, such as false positive findings and overdiagnosis, and the lack of evidence that increased detection improves breast cancer mortality rates [19, 20]. In the German national screening programme, additional examinations, such as US, are typically used only for further assessment of mammographically detected or clinically ambiguous findings [21]. Moreover, women are generally not informed about their breast density or its implications for diagnostic accuracy.

Potential barriers to the introduction of supplemental US examinations as part of mammography screening include a lack of resources (e.g., experienced screeners), anticipated increases in false positive findings leading to additional tests such as biopsies or further imaging, associated with psychological stress for screening clients, and higher workloads for healthcare providers, alongside rising costs for the healthcare system [22]. Therefore, feasibility relies on a careful selection of women at the highest risk among those with dense breasts [23, 24].

Although current literature explores the benefits of additional screening techniques in addition to mammography, little is known about the capacity of national mammography screening programmes to routinely integrate US for women with dense breast tissue. One objective of the DIMASOS 2 study (Density-Indicated Mammographic-Sonographic Breast Cancer Screening) was therefore to investigate the practicality of incorporating US as an adjunct to mammography for women with dense breasts. To this end, a qualitative study utilising semi-structured telephone interviews was conducted to gather insights from health care providers (physicians, radiographers) and screening clients at six German public mammography screening centres that had implemented automated breast density measurement and additional handheld US for eligible clients. The study aimed to explore the perceptions of healthcare professionals and screening clients regarding women’s awareness of dense breast tissue, its impact on mammography effectiveness, and the overall acceptability and feasibility of incorporating supplemental US.

## Methods

This explorative qualitative study was part of DIMASOS 2, a prospective controlled multicentre mixed-methods study in which breast density was automatically measured during mammography screening. Between 2020 and 2024, 16 German screening sites participated. Qualitative telephone interviews were conducted between November 2020 and November 2021 with healthcare professionals and screening clients identified as having dense breast tissue at six of the participating centres. Breast density was assessed using Densitas®software [14]. Women with dense breasts were offered US in addition to mammography if their breast density was classified as either very dense or heterogeneously dense, using a threshold that approximately included the upper 18% of women by breast density. The additional US was offered within one week following the mammogram and, in some cases, was performed immediately after the mammographic screening. All US examinations were performed by experienced physicians.

The study adhered to the guidelines for Reporting Qualitative Research (SRQR) [25] and was reviewed by the ethics committee of Lübeck University (reference number: 19–361). Participation was voluntary and written informed consent was obtained before the interview. Researchers not directly associated with the professionals and clients conducted the telephone interviews, which followed a semi-structured interview guide.

### Participants

#### Mammography screening clients

The sample consisted of 22 clients who attended the German mammography screening programme and were identified as having dense breast tissue. Eligible participants were between 50 and 69 years old. Recruitment was conducted by the radiographer after measuring the women’s breast density. Each woman received a brochure containing information about breast density and its effects on mammography screening, along with an invitation to participate in a voluntary telephone interview.

#### Professionals

Interviews were conducted with 7 physicians (gynaecologists or radiologists), including 4 heads of the participating screening centres, and 2 radiographers. All physicians were experienced mammography screeners.

### Data collection

Based on a previous literature review, two flexible semi-structured interview guides were developed: one for the physicians and staff and one for the clients. The clients’ guide covered topics such as perceptions of breast density, its impact on the accuracy of the screening programme, and individual experiences with the additional examination. The interview guides used in this study are provided in the supplementary materials (S1, S2).

The interviews with women began with the question of whether this was the first time they had learnt that their breast tissue was dense and what thoughts and feelings they had about this. They were then asked about their experiences with the additional US. Finally, they were interviewed about their perceptions of the effectiveness of mammography screening and whether their opinion had changed due to the information provided about dense breasts.

The interview guide for physicians and staff assessed their perceptions regarding women’s awareness of breast density and its impact on the sensitivity of the mammography screening programme. Additionally, the feasibility of incorporating the additional US into routine screening was evaluated, including its advantages and disadvantages.

All interviews were recorded and transcribed verbatim, with deidentification of places and names.

### Data analysis

Data analysis was performed using qualitative content analysis [26] using the open access application QCAmap (Qualitative Content Analysis Program) [27]. Initially, the interview data from professionals and clients were analysed independently by two researchers (PG & SE). Due to the explorative approach of this study, the material was coded by extracting inductive categories from the interview data.

**Fig. 1:**
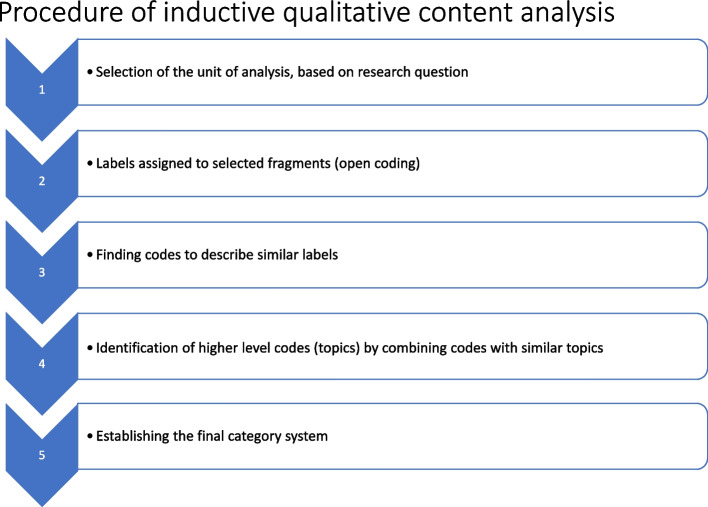
Inductive categorisation process

To create coding schemes for (a) the professionals and (b) the clients’ data, both researchers began by analysing two interviews from each dataset. This process involved open coding**,** where text fragments that were meaningful and relevant to the research question were identified and labelled. These fragments could be sentences, phrases, or words. Based on the content analytical rules for inductive category formation, a level of abstraction was defined at which the categories were to be formulated. Additionally, the coding unit, defined as the smallest codable component of the text that can be assigned a code, was established. After the initial coding, the codes were analysed to identify patterns and relationships among the data. Similar codes were then grouped to form more abstract categories. This process was iterative and categories were refined and modified constantly to increase clarity and reduce overlap. A category system, containing the inductively developed codes was then created. The category systems used in this study are provided in the supplementary materials (S3, S4). Regular meetings were held to discuss themes, codes and interpretations, to ensure consistency and reliability and to ultimately agree on one single coding scheme for professionals and another for clients. The remaining interviews were then coded using these finalised schemes (Fig. 1).

## Results

A total of 31 telephone interviews were conducted: 22 with mammography screening clients, 7 with physicians (including four heads of screening units), and two with radiographers. Participants were drawn from six different German screening units: Tübingen, Hildesheim, Mainz, Bad Langensalza, Königs Wusterhausen, and Alzey). The average age of the screening clients was 58 years, the average age of the professionals was 55 years. The duration of the interviews ranged from 20 to 90 min (Table 1).

**Table 1:** Characteristics of the interviewees and interview duration

Characteristics	N
Professionals	
Gender	
Female	6
Male	3
Speciality	
Head of a screening site	5
Screening doctor	2
Radiographer	2
Interview time in minutes (median)	35
Age (median)	55
Screening clients	
Interview time in minutes (median)	15
Age (median)	58

### Awareness and perceptions of breast density

#### Clients

Among the 22 women surveyed, 11 learnt for the first time that their breast tissue was dense. One client explained: *“The nurse explained that dense glandular tissue had been detected and that I would probably know that. That irritated me a bit because I had never heard of it before. I've only heard that there are milk breasts and meat breasts. That was the only information I’ve ever heard.” (Screening client #7*).

Almost all women recognised that breast density can complicate mammogram readings after reading the study information. One client stated: *„Well, sometimes you might not be able to see an incipient lump as easily in dense tissue as if it were looser.” (*Screening Client #18). Among those who were aware of their dense breasts, three had already undergone US for further assessment: *“My gynaecologist had diagnosed it once and, if I remember correctly, she had already done an US somehow. But I don't think these examinations are free, so I was actually quite happy that I was able to have it as part of the study, for free."* (Screening Client #13) For others, this information has had no consequences so far: *"Actually, it hasn't had any consequences so far. The only thing – you just worry when you have so much tissue. That if there really is something, you can't see it."* (Screening Client #20). Another noted: *“I kind of already knew I had it. I just didn't realise, what effects it could have.”* (Screening Client #9) None of the interviewed women mentioned dense breast tissue as an independent risk factor for breast cancer.

Most women indicated that the information about dense breast tissue did not overly concern them. One commented: *„Oh, I have taken note of that. And I have also taken note that there are certain uncertainties when it comes to early detection. But to be honest, that didn't worry me."* (Screening Client #10).

#### Professionals

The professionals had varied views on women’s knowledge of dense breast tissue. One expert suggested that women are generally aware of the topic: *„It’s not really an issue. Most women know.”* (Professional #3). Others, however, estimated that 25 to 40 percent truly understand its implications: *“I think that one quarter already know what it means, but the rest doesn’t really know."* (Professional #6) One physician expressed scepticism about general knowledge: *“More likely, there is no knowledge at all”.* (Professional #1).

### Impact of breast density on public mammography screening

#### Clients

Interviewed screening clients expressed great confidence in public mammography screening and valued its organisation: *“Everything was very professional, everyone was very friendly, very approachable.”* (Screening Client #10). The diagnosis of dense breast tissues did not diminish their positive attitude towards mammography’s effectiveness. Some appreciated the additional US, feeling it enhanced their safety: *“I felt even more valued …that the development has gone so far that they see a way to minimise the risk of missing breast cancer even more."* (Screening Client #7).

However, a few women indicated a preference for US alone to avoid the discomfort and radiation exposure associated with mammograms: *"Unless it turns out that mammography doesn't do anything for me anyway and US is much better. Then I'd probably go for an US, because there's no radiation exposure."* (Screening Client #13).

#### Professionals

All professionals recognised the public mammography screening programme as very effective and well organised: *“I am convinced by the evidence, yes. But if you can reduce that (the mortality) a bit more, then of course I'll be right behind you, sure.”* (Professional #5). They agreed that dense breast tissue poses challenges in mammogram interpretation: *“There are perhaps 10 percent of cases where we have really dense breasts … that's where we really have a problem with mammography.”* (Professional #9).

Most professionals felt that for the small number of women with dense breasts, additional examinations like US are required to reduce interval cancers and breast cancer mortality: *“Well, I suffer a lot from interval cancers, although I know that no screener in Germany or anywhere else can get by without interval cancers.”* (Professional #4). Some professionals pointed out that standards should be developed to enhance screening quality: *“Mammography screening, as the name suggests, has so far only been based on mammography. That is not enough, as our critics say. And I agree with the critics.”* (Professional #7).

A few professionals suggested digital breast tomosynthesis (DBT) as an alternative to US: *“So if the data situation shows it, based on studies, I would be completely open to tomosynthesis.”* (Professional #5). Conversely, some expressed their concerns, stating that the current screening process was sufficient, fearing that additional examinations would merely lead to false positive findings*: "I think the current screening programme is very good and I believe that the additional sonographic examination for dense breasts is of no use at all. Yes? And especially as individual skills play a relatively large role. So, I certainly didn't initiate any further unnecessary measures, i.e. false positives, as a result of these additional examinations. That would be a real harm.”* (Professional #3).

Concerns about overdiagnoses were also expressed by others: *“So everything we do, we're getting better and better. We're finding smaller and smaller carcinomas. Doesn't mean that we're reducing mortality. If we find the smaller ones, it doesn't mean that women die less frequently. No? It may also be that an overdiagnosis is being produced.”* (Professional #7). One physician worried that public mammography screening could lose its good reputation if the uncertainties surrounding dense breasts and the limitations of mammography in dense breasts were communicated more widely: *“In general, I have a little question mark for myself because women might think: 'Oh, they've realized now that mammography doesn't work."* (Professional #4).

### Feasibility of handheld breast US as a supplemental examination for women with dense breasts

#### Clients

All women indicated that the US examination itself was not inconvenient, though scheduling posed challenges. US was sometimes offered on the same day as mammography, but sometimes several days passed before the US occurred. Two women described the waiting period as stressful:”Of *course I was nervous, but always with the hope that there wouldn't be anything there. But it was an uneasy feeling.” (*Screening Client #6). For this reason and for reasons such as a long journey to the screening unit, most women preferred to be offered the additional examination immediately after the mammogram: *"It would be nice, of course, if you could do it in one day. I'm lucky that I'm at home at the moment and that I had time.”* (Screening Client #1).

#### Professionals

Some screening professionals highlighted challenges in offering US to women living in rural areas: *“Well, and then, look, we have a mobile unit, the woman who is being x-rayed lives really far away from us, sometimes 50 kms, so you would have to say: ‘You have dense breasts. We can offer you an US, but we can't do it on our trailer.’ That would mean these women would have to come to the screening unit for the US. Yes? That's, logistically, that's quite a word."* (Professional #5).

One professional pointed out the advantages of US, stating: *“Well, I actually think that's the most sensible thing to do, because there's no radiation exposure and it's virtually low-threshold, anyone can do it anywhere, there are no contraindications at all.”* (Professional #4). However, the need for experienced staff to ensure the effectiveness of the measure was also emphasised: “*It’s not nothing. It doesn't do any good if you put someone in there to do it and they don't see the carcinoma, yes?"* (Professional #4). In addition to having skilled screeners, the implementation of supplemental US also requires significant resources, including time and room capacity. For most professionals, this turned out to be the main barrier to incorporating US into their daily routine: *“We wouldn't be able to manage that in terms of structure. That would actually destroy our structures.”* (Professional #2).

Concerns were raised about the limited additional detection of tumours through US, with several professionals expressing surprise at the low number of additional findings: “*To be honest, I had expected that we would find more in addition. Just from a purely emotional point of view, I always think: "It's strange that we fish out so little extra."* (Professional #2), or expressed by another professional: *“The number of cases that we are picking out that have a carcinoma that can only be seen by US is frighteningly small.”* (Professional #7). Conversely, another physician described that she was relieved when she learnt that mammography does not miss a large number of tumours in dense breast tissue:*”So we're aware of all the things you can't see in dense tissue, and we just held our breath with dense tissue in the sense of hopefully not missing anything. And I have to say that I'm a bit more relaxed now that I've done a lot of dense tissue US and realised that there's not as much to miss as you always think."* (Professional #4).

The link between additional breast US examinations and false-positive findings, which could lead to unnecessary biopsies, was also discussed: *“Yes, the disadvantage is an increase in biopsies, and the programme must be evaluated, and it must ultimately reduce mortality, but here's the thing: this must be achieved by minimising the psychological and physical burden on the women, the clients, the patients.”* (Professional #7). Some physicians estimated what kind of tumours the additional US examination could possibly detect: *“I think we could find eight per thousand instead of six per thousand. What kind? I think it will be small, not very aggressive cancers. Whether that really effects overall survival, I don't know.”* (Professional #5).

Suggestions were made for conducting US examinations between screening rounds to better detect interval carcinomas: *“You might have to do the US between, you know, not directly with the mammogram, but between two examinations, that's practically a year later."* (Professional #5). Concerns about involving local physicians for US examinations were raised due to potential variability in quality: *“If the gynaecologists are informed of the density of the breast, sonographic examinations start in the wrong hands and further unnecessary examinations and biopsies, are carried out. This is exactly what they wanted to prevent by introducing the (screening) programme.”* (Professional #3).

Moreover, performing US examinations requires intense focus: *“… after 30 scans or so I'm actually exhausted and I realise I can't make good decisions anymore."* (Professional #4). This led some professionals advocate for automated breast US screening: *“I think we should consider using automated breast scans. It will standardise the procedure.”* (Professional #8). On the contrary, some professionals were sceptical about automated US: *“The demos I've seen, I immediately get totally tired of looking at these moving images. For an experienced screener*, *it's more time-efficient to do the US directly and finish it than to sit in front of a film for 5 min.” (*Professional #4). Concerns about the costs associated with automated systems were also highlighted: *“… it's technically so complex and also so expensive that it can't be implemented on a broad scale."* (Professional #9).

Almost all professionals acknowledged the complexity of managing women with dense breast tissue, expressing the significant responsibilities involved: *“You have a great responsibility towards the women. And you have a great responsibility towards the cost bearers and therefore towards society. It's all very difficult.”* (Professional #7).

## Discussion

This study describes the impact of dense breast tissue on mammography and the feasibility of handheld US in addition to mammograms in the German public mammography screening programme for women identified of having dense breast tissue. The experiences of professionals and screening clients of six screening units were investigated by conducting and analysing semi-structured telephone interviews with clients, physicians and staff. Three main topics were covered by the investigation: (1) Awareness and perceptions of breast density, (2) Impact of breast density on the accuracy of mammography screening, (3) Feasibility of handheld breast US as supplemental examination for mammography screening clients with dense breasts.

In our study, approximately half of the women surveyed had been informed about having dense breasts tissue prior to participating in the study. For most of these women, this information had not led to significant consequences, such as additional examinations. Moreover, none were aware that dense breast tissue is an independent risk factor for developing breast cancer. However, when explained, all surveyed women understood that breast density can mask cancer on mammograms. This finding is consistent with Beidler et al. [28] who investigated perceptions of high breast density in US women and found that nearly all participants understood that breast density can mask cancer on a mammogram, but most did not recognise breast density as an independent risk factor for breast cancer.

In Germany, breast density is not routinely mentioned in the mammography report, and women do not usually see a physician during the screening examination. Therefore, women with dense breasts are often led to believe that mammography is just as effective for them as it is for women with less dense breast tissue. This reflects a lack of information that could negatively affect trust in the mammography screening programme and the doctor-patient relationship, which, in the case of a higher public awareness, could lead to an overall lower participation rate in mammography screening programmes.

Some professionals interviewed expressed reservations regarding informing screening clients of dense breast tissue and its potential implications for mammography. They were concerned that this might contribute to a negative perception of the screening programme and lead to additional examinations being conducted by less experienced doctors. A study performed in the UK [29], which investigated the perspectives of radiologists and breast surgeons on informing women about dense breasts tissue, found that 60% of surveyed physicians did not routinely disclose this information during discussions of screening mammography results. This was largely attributed to the absence of clear, evidence-based guidelines. The non-disclosure of breast density may limit women’s ability to fully consider this factor when making decisions about continued participation in screening programmes or the potential use of supplementary examinations. Awareness of dense breast tissue has been shown to increase the willingness to undergo routine and supplemental breast cancer screening, which may facilitate earlier detection [30, 31]. Although direct evidence that awareness prompts women to seek medical advice when symptoms arise is limited, increased awareness may encourage more proactive health behaviours. However, in the absence of consensus guidelines on how to manage dense breast tissue and the role of additional examinations, physicians face challenges in offering consistent, evidence-based recommendations. As a result, screening practices can differ significantly across healthcare providers, which may lead to uncertainty among both patients and professionals and complicate the process of informed decision-making.

Interviewed screening clients generally appreciated the additional US, with some reporting an increased feeling of safety as a result. A few women expressed uncertainty whether mammograms were useful for them at all, indicating a preference for regular US examinations over mammograms due to concerns about radiation exposure and the discomfort associated with breast compression. The absence of clear, evidence-based guidelines limits the information that can be provided to clients with such concerns, which may increase the risk of misunderstandings or incomplete communication. Some screening clients found the information regarding dense breast tissue to be confusing, and experienced the waiting period between the mammogram and the subsequent US unsettling, leaving some of them worried about their health.

This study showed that women informed about their breast density require clear and comprehensive information regarding its significance, including the general effectiveness of mammography in cancer detection and the supplementary nature of additional examinations. Previous research indicates that breast density notifications may cause anxiety and confusion for some women concerning the implications and subsequent consequences [32–35]. Therefore, it is essential for mammography screening centres to provide accurate information and ensure that any supplemental examinations are carried out by qualified specialist. Without this, there is a risk that information may be misunderstood, and additional procedures may be carried out by less experienced physicians, potentially leading to unnecessary harms and costs, a concern frequently raised by the experts interviewed. The interviewed experts agreed that performing US for breast cancer detection requires significant expertise, and currently, there is a shortage of experienced personnel and sufficient time to incorporate this into routine screening.

Professionals estimated that dense breast tissue complicates mammogram evaluation in approximately 10% of screening clients. Current literature also describes the loss of diagnostic sensitivity in public breast cancer screening programmes for women with dense breasts [7, 8, 11, 12]. Therefore, some of the professionals considered that the sensitivity of the programme needs to be improved by incorporating additional examinations for women at risk. Conversely, other interviewees considered the existing screening approach sufficient, expressing concerns that additional screening could increase false-positive results, overdiagnosis, and overtreatment. Opinions also varied regarding the preferred method for additional screening, with some favouring DBT and others promoting automated US over handheld US. Similarly, two US studies reported differing views among radiologists and other physicians about the impact of dense breasts tissue on mammography and the recommendations for supplemental screening for women at risk [36, 37]. Both studies emphasised the need for evidence-based guidelines to improve patient management and reduce unnecessary healthcare costs.

Regarding the feasibility of US, scheduling difficulties were reported as the main challenges for screening clients. Nevertheless, most women surveyed felt that the gains in diagnostic accuracy and perceived sense of safety outweighed these drawbacks. A key concern was that US cannot be offered equally to all women. In rural areas, for instance, mammography is often offered on mobile units, which lack space to accommodate US equipment and the presence of qualified staff to perform the supplemental US. As a result, women in these regions would need to travel to the nearest urban screening centre, where sufficiently trained staff and facilities are available.

Most professionals surveyed considered handheld US a generally practical method for improving diagnostic accuracy in women with dense breast tissue. However, all experts reported significant challenges in integrating US into routine screening due to limited capacity and ongoing staff shortages. Key barriers included the need of additional resources, such as room capacities, certified staff with appropriate training, and extra time. Furthermore, experts noted that a large number of US examinations were often required to detect a single additional tumour not identified by mammography. As a result, enthusiasm for conducting US declined over time, particularly given that the procedure is more time-consuming than double reading of mammograms and led to relatively few additional breast cancer detections. Other studies have also described that handheld US requires experienced staff, making US accuracy highly dependent on the skill of the examiner [23].

Some professionals estimated that additional US might primarily detect slow-growing, small tumours. Some of the experts expressed concerns about a potential increase in false-positive findings, which could negatively impact the balance of benefits and harms within the public mammography screening programme. They pointed out that a reduction in mortality attributable to US must be proven before its permanent integration into the screening programme. A recently published review concluded that although additional US increases the cancer detection rate, it also results in a significant increase in false-positive findings. Therefore, it remains to be seen whether the additional cancer detection leads to reduced mortality and morbidity, or merely contributes to increased screening-related harms [3].

### Strengths and limitations

This study presents a retrospective qualitative analysis of the experiences of both screening clients and healthcare professionals regarding the implementation of handheld breast US in women with dense breast tissue. The retrospective study design may introduce recall bias. This is particularly true for the screening clients, as the time between US and interview was not systematically recorded for all participants, limiting the ability to fully assess the potential impact of recall bias. Furthermore, the study sample included only screening clients with benign or normal findings who consented to the additional US; women diagnosed with breast cancer were not interviewed, which may limit the generalisability of the findings, potentially skewing the results toward a more positive perception of the additional examination. For many women, the experience of undergoing additional examinations, such as biopsies following a positive US result, can be a source of significant stress and anxiety. These negative feelings may persist even when final result turns out negative. Such an experience may lead to labelling bias and may contribute to a perception of harm rather than benefit, which may discourage some women from participating in future screenings. Another limitation of the study is the relatively small number of expert interviews conducted (n = 9), which may lead to a sample skewed toward the perspectives of heads of screening centres (n = 4). Their views may not fully represent those of other staff members involved in the screening process. However, the inclusion of physicians with programme responsibility may also be considered a strength, as their insights reflect authority in programme decisions and a comprehensive understanding of the implementation and management of the screening programme. A further limitation is the lack of detailed sociodemographic information beyond participants’ date of birth and address. This missing data restricts our ability to fully contextualise the findings and explore how factors such as educational background, participation history, or geographic setting might have influenced participant responses.

A strength of the study is the insight it provides into the perceived feasibility of handheld US within the context of six mammography screening sites in Germany. As the integration of additional US to mammography for women with dense breast tissue is being considered as a potential strategy to increase diagnostic accuracy, these findings contribute valuable information on the practical considerations and challenges associated with implementing this approach. Given the current lack of evidence on this subject, this study offers important insights.

## Conclusion

Breast US in addition to mammography was generally perceived positively by both health professionals and clients, as it does not require additional radiation exposure and is theoretically straightforward to provide. However, all professionals interviewed reported significant challenges in introducing handheld US for women with dense breast tissue within public mammography screening. The primary barriers identified were limited time and a shortage of experienced screeners. While professionals largely agreed on the importance of improving screening accuracy for women with dense breasts, opinions varied regarding the optimal approach to achieve this, alongside concerns about an increase in false positive findings.

The absence of clear consent procedures on additional screening contributes to a lack of information provided to women at risk. This limits their ability to take proactive measures for breast health, which could support early detection and improve outcomes. However, this complex issue should not lead to the development of opportunistic screening of uncertain quality. Developing targeted, evidence-based guidelines for breast cancer screening in women with dense breasts is essential to address these specific needs while preserving public confidence in national mammography screening programmes.

## Supplementary Information

Supplementary Material 1.

## Data Availability

The anonymised interview transcripts used and/or analysed in this study are available upon request from the corresponding author.

## Abbreviations

DBT: Digital breast tomosynthesis
DIMASOS2: Density-Indicated Mammographic-Sonographic Breast Cancer Screening
QCAmap: Qualitative Content Analysis Program
US: Ultrasound

## References

[1] Broeders M, et al. The impact of mammographic screening on breast cancer mortality in Europe: a review of observational studies. J Med Screen. 2012;19(Suppl 1):14–25. 22972807 10.1258/jms.2012.012078

[2] Marmot MG, et al. The benefits and harms of breast cancer screening: an independent review. Br J Cancer. 2013;108(11):2205–40. 23744281 10.1038/bjc.2013.177 PMC3693450

[3] Glechner A, et al. Mammography in combination with breast ultrasonography versus mammography for breast cancer screening in women at average risk. Cochrane Database Syst Rev. 2023;3(3):Cd009632. 36999589 10.1002/14651858.CD009632.pub3 PMC10065327

[4] Nelson HD, et al. Harms of breast cancer screening: systematic review to update the 2009 U.S. preventive services task force recommendation. Ann Intern Med. 2016;164(4):256–67. 26756737 10.7326/M15-0970

[5] Häberle L, et al. Mammographic density is the main correlate of tumors detected on ultrasound but not on mammography. Int J Cancer. 2016;139(9):1967–74. 27389655 10.1002/ijc.30261

[6] Girardi V, et al. Breast ultrasound in 22,131 asymptomatic women with negative mammography. Breast. 2013;22(5):806–9. 23558244 10.1016/j.breast.2013.02.010

[7] Boyd NF, et al. Mammographic density and the risk and detection of breast cancer. N Engl J Med. 2007;356(3):227–36. 17229950 10.1056/NEJMoa062790

[8] Weigel S, et al. Digital mammography screening: sensitivity of the programme dependent on breast density. Eur Radiol. 2017;27(7):2744–51. 27822617 10.1007/s00330-016-4636-4

[9] Wanders JO, et al. Volumetric breast density affects performance of digital screening mammography. Breast Cancer Res Treat. 2017;162(1):95–103. 28012087 10.1007/s10549-016-4090-7 PMC5288416

[10] Payne NR, et al. Breast density effect on the sensitivity of digital screening mammography in a UK cohort. Eur Radiol. 2025;35(1):177–87. 39017933 10.1007/s00330-024-10951-w PMC11631811

[11] Gordon PB. Breast density and risk of interval cancers. Can Assoc Radiol J. 2022;73(1):19–20. 34482760 10.1177/08465371211030573

[12] Advani SM, et al. Association of breast density with breast cancer risk among women aged 65 years or older by age group and body mass index. JAMA Netw Open. 2021;4(8):e2122810. 34436608 10.1001/jamanetworkopen.2021.22810 PMC8391100

[13] Kerlikowske K, et al. Combining quantitative and qualitative breast density measures to assess breast cancer risk. Breast Cancer Res. 2017;19(1):017–0887. 10.1186/s13058-017-0887-5 PMC5567482 28830497

[14] Abdolell M, et al. Utility of relative and absolute measures of mammographic density vs clinical risk factors in evaluating breast cancer risk at time of screening mammography. Br J Radiol. 2016;89(1059):21. 10.1259/bjr.20150522 PMC4986486 26689094

[15] Bodewes FTH, et al. Mammographic breast density and the risk of breast cancer: a systematic review and meta-analysis. Breast. 2022;66:62–8. 36183671 10.1016/j.breast.2022.09.007 PMC9530665

[16] Corsetti V, et al. Evidence of the effect of adjunct ultrasound screening in women with mammography-negative dense breasts: interval breast cancers at 1 year follow-up. Eur J Cancer. 2011;47(7):1021–6. 21211962 10.1016/j.ejca.2010.12.002

[17] Wu T, Warren LJ. The added value of supplemental breast ultrasound screening for women with dense breasts: a single center Canadian experience. Can Assoc Radiol J. 2022;73(1):101–6. 34134531 10.1177/08465371211011707

[18] Sprague BL, et al. Performance of Supplemental US Screening in Women with Dense Breasts and Varying Breast Cancer Risk: Results from the Breast Cancer Surveillance Consortium. Radiology. 2024;312(2):e232380. 39105648 10.1148/radiol.232380 PMC11366666

[19] Isautier JMJ, et al. Clinical guidelines for the management of mammographic density: a systematic review of breast screening guidelines worldwide. JNCI Cancer Spectr. 2024;8(6). 10.1093/jncics/pkae103 PMC11578290 39392432

[20] Henderson JT, et al. Screening for breast cancer: evidence report and systematic review for the US preventive services task force. JAMA. 2024;331(22):1931–46.38687490 10.1001/jama.2023.25844

[21] Albert US, et al. 2008 update of the guideline: early detection of breast cancer in Germany. J Cancer Res Clin Oncol. 2009;135(3):339–54. 18661152 10.1007/s00432-008-0450-y PMC12160260

[22] Lauby-Secretan B, et al. Breast-cancer screening–viewpoint of the IARC Working Group. N Engl J Med. 2015;372(24):2353–8. 26039523 10.1056/NEJMsr1504363

[23] Lobig F, et al. Performance of supplemental imaging modalities for breast cancer in women with dense breasts: findings from an umbrella review and primary studies analysis. Clin Breast Cancer. 2023;23(5):478–90. 37202338 10.1016/j.clbc.2023.04.003

[24] Rebolj M, et al. Addition of ultrasound to mammography in the case of dense breast tissue: systematic review and meta-analysis. Br J Cancer. 2018;118(12):1559–70. 29736009 10.1038/s41416-018-0080-3 PMC6008336

[25] O’Brien BC, et al. Standards for reporting qualitative research: a synthesis of recommendations. Acad Med. 2014;89(9):1245–51. 24979285 10.1097/ACM.0000000000000388

[26] Mayring P. Qualitative content analysis: a step-by-step guide. London: SAGE; 2022.

[27] Fenzl T, Mayring P. QCAmap: eine interaktive Webapplikation für Qualitative Inhaltsanalyse. Z Soziol Erzieh Sozialisation ZSE. 2017;37:333–40.

[28] Beidler LB, et al. Perceptions of breast cancer risks among women receiving mammograph screening. JAMA Netw Open. 2023;6(1):e2252209. 36689223 10.1001/jamanetworkopen.2022.52209 PMC9871800

[29] Varghese J, et al. Breast density notification: current UK national practice. Clin Breast Cancer. 2022;22(1):e101–7. 34099394 10.1016/j.clbc.2021.04.013

[30] Tran ATN, et al. Impact of awareness of breast density on perceived risk, worry, and intentions for future breast cancer screening among Korean women. Cancer Res Treat. 2021;53(1):55–64. 32810929 10.4143/crt.2020.495 PMC7812003

[31] Santiago-Rivas M, et al. Breast density awareness and knowledge, and intentions for breast cancer screening in a diverse sample of women age eligible for mammography. J Cancer Educ. 2019;34(1):90–7. 28808894 10.1007/s13187-017-1271-y PMC5812844

[32] Dench EK, et al. Confusion and anxiety following breast density notification: fact or fiction? J Clin Med. 2020;9(4). 10.3390/jcm9040955 PMC7230559 32235552

[33] Huang S, et al. The impact of mandatory mammographic breast density notification on supplemental screening practice in the United States: a systematic review. Breast Cancer Res Treat. 2021;187(1):11–30. 33774734 10.1007/s10549-021-06203-w

[34] Nickel B, et al. The impact of breast density information or notification on women’s cognitive, psychological, and behavioral outcomes: a systematic review. J Natl Cancer Inst. 2021;113(10):1299–328. 33544867 10.1093/jnci/djab016 PMC8486329

[35] Isautier JMJ, et al. The impact of breast density notification on psychosocial outcomes in racial and ethnic minorities: a systematic review. Breast. 2024;74:103693. 38430905 10.1016/j.breast.2024.103693 PMC10918326

[36] Lourenco AP, DiFlorio-Alexander RM, Slanetz PJ. Breast density legislation in New England: a survey study of practicing radiologists. Acad Radiol. 2017;24(10):1265–7. 28495213 10.1016/j.acra.2017.03.009

[37] Maimone S, McDonough MD, Hines SL. Breast density reporting laws and supplemental screening-a survey of referring providers’ experiences and understanding. Curr Probl Diagn Radiol. 2017;46(2):105–9. 27289137 10.1067/j.cpradiol.2016.05.001

