# Supplementary material for: Optimising breast cancer screening in national mammography screening centres: challenges and insights on implementing additional ultrasound for women with dense breast tissue — a qualitative study
Source: BMC Cancer. 2025 Oct 31;25:1684. doi: 10.1186/s12885-025-15145-1 (PMC12579404; doi:10.1186/s12885-025-15145-1)
Supplement: Supplementary file 1 — Supplementary Material 1. [file 12885_2025_15145_MOESM1_ESM.docx]

**S1: Interview guide experts**

Before the interview begins, information about the name, age and profession of the interviewee is obtained. In addition, the interviewer should ensure that the environment is free of noise so that the interview can be conducted in a quiet and trustworthy situation. The interviewees are informed about the objectives of the study; if there are any unanswered questions, these should be clarified at this stage. The interviewer obtains informed consent for participating in the study. The interviewees are then informed that the interview will be recorded, that the recording will now begin and whether they agree to the recording.

| Key question | What is to be asked? | Additional questions |
| --- | --- | --- |
| You work as a doctor / radiographer in a mammography screening unit. Could you please describe a typical working day for you? As I have no experience in this field myself, it would help me to better imagine the daily routine in mammography screening. | Individual experiences  Which tasks have priority  Time and workload | How was your day today, for example? |
| Are there any special characteristics regarding the women who come to your screening unit for examination? Is your region, for example, more rural or urban? | What are the characteristics of the screening unit (urban, rural, mixed)? What is the age structure? | What is the age structure?  Are there women who may feel reluctant to participate in the mammography screening? What reasons might there be for women not to participate? |
| Are there any particular challenges for you as a doctor / radiographer and your work in mammography screening? | Challenges | Are there particular medical challenges? |

| Your screening unit is part of the DIMASOS2 study. You screen women with dense breast tissue using ultrasound in addition to mammography.  How relevant is the topic of dense breast tissue for your daily work? | Thoughts and perceptions on the topic  Assessment of evidence | Do you think the topic has been neglected so far? |
| --- | --- | --- |
| Has anything changed for you and your work organisation / every day working life as a result of participating in the study? | What challenges does the additional examination entail? | Do you need additional time to provide information for the screening participants?  How time consuming are these additional conversations? The topic is relatively complex, how well can it be explained to the participants?  How much prior knowledge do the screening participants generally have?  Are there women who refuse the additional examination? What reasons do these women give?  How often are further examinations, such as biopsies, MRI required after the additional ultrasound?  How often do false-positive findings occur, in your opinion? |
| How do you think the additional ultrasound examination will affect the detection of breast cancer? | Assessment of the evidence  Assessment of the additional benefits | What alternatives to this approach could be considered? What do you think of these alternatives?  Are there types of tumours that could be missed by ultrasound? Perhaps because they cannot be visualised well with ultrasound?  Do you also see possible disadvantages that may arise from the additional examination? |

| How do you assess the evidence of the traditional mammography screening? | Assessment of the effectiveness  Assessment of side effects | Are there gaps in the evidence?  How effective is mammography screening in your opinion?  What could be improved?  Are certain populations groups excluded, and is this problematic? (E.g. women under 50 and over 70 years) |
| --- | --- | --- |
| What conditions would have to be in place to be able to offer the additional ultrasound examination to affected women in the future? | Ask about potential burdens as well as benefits of combined screening | Are the basic conditions in the screening units in place?  What is missing? Equipment? Personnel? |
| What do the study participants generally think of the additional ultrasound examination? | How is the offer generally accepted? | Is there any criticism of the additional examination?  Are there any other particularities? (e.g. concerns / fears of the participants, waiting times) |
| Do you take part in mammography screening yourself (female interviewees)? | Slow topic change. Conclude the interview | What reasons led to your decision to participate in the screening / not participate in the screening? |
| What would you advise your female relatives or friends: to go for the screening or not? | This question can also be asked to men |  |
| Is there anything you would like to change or optimise when you think about mammography screening? |  | What are the advantages and disadvantages of screening? |
| Thank you for taking the time and sharing your experiences with us.  Maybe you would like to add or comment on something? Are there any points you would like to address that haven't been mentioned yet? |  |  |

**S2: Interview guide screening clients**

Before the interview begins, information about the name and age of the interviewee is obtained. In addition, the interviewer should ensure that the environment is free of noise so that the interview can be conducted in a quiet and trustworthy situation. The interviewees are informed about the objectives of the study; if there are any unanswered questions, these should be clarified at this stage. The interviewer obtains informed consent for participating in the study. The interviewees are then informed that the interview will be recorded, that the recording will now begin and whether they agree to the recording.

| Key question | What is to be asked? | Additional questions |
| --- | --- | --- |
| Thank you for taking the time. How are you today? |  |  |
| You have recently participated in breast cancer screening. It was found that the tissue in your breast is particularly ‘dense’, is that correct? | What prior knowledge exists?  Was the interviewee aware that she has high breast density? | IS this the first time you have heard that you have dense breasts?  If you have known for a longer time, what consequences has this had for you so far?  Have you heard before that there is dense and less dense breast tissue?  In your opinion, what impact does dense breast tissue have on mammography and the diagnosis of breast cancer? |

| I would like to come back to the situation when you were told that your breast tissue is dense. Can you please describe this in more detail? What was that like? | Experience of the diagnosis, feelings.  Quality of the information from the perspective of the interviewee (comprehensibility, formal structure of the conversation).  Is there prior knowledge (e.g., from the media)? | Who told you that you have dense breast tissue?  What did the doctor say?  How were you explained what this means and why further examination is recommended?  How did you experience that? What feelings did you have? |
| --- | --- | --- |
| In addition to the mammogram, an ultrasound was offered for further clarification. Has the ultrasound already been carried out? | Has the ultrasound already been performed?  Is the final result already known?  Have any further examinations (e.g., biopsy, MRI) been carried out? | How long did it take until the additional ultrasound was carried out?  Who did the ultrasound?  How was the examination for you? How did you feel about the examination?  What were the results of the examination? |
| What happened after the ultrasound examination? | Were any additional examinations conducted besides the mammogram and ultrasound? | Were there any follow-up examinations (e.g., a biopsy, MRI)?  What information did you have about these examinations?  What experiences did you have during the follow-up examinations?  What was done? And why was it done?  How long did you have to wait for the examinations?  Is there already a final result? If so, what is it?  If the result is positive: Has the treatment plan already been discussed with you?  Do you find the treatment suggestions reasonable for you?  If there is still no result:  How long will it be before you are informed of the results? |

| As part of your participation in the study, were there any stresses or burdens that you were not prepared for? | Enquire about possible burdens but also advantages of combined mammography / sonography | I mean burdens, for example, due to the uncertainty or the waiting time until the result? |
| --- | --- | --- |
| Have you received any recommendations for future screening mammograms? |  | Will you continue to participate in mammography screening in the future?  Has your participation in the study changed your attitude towards screening? |
| I have a general question: For how many years have you been going for mammograms? | Slowly change the topic. Conclude the interview.  Enquire about possible previous stress / findings.  Assessment of effectiveness.  Assessment of side effects. | What are your reasons for participating in breast cancer screening?  Is there a particular reason or recommendation?  Has there ever been a special event during screening mammography?  How effective do you think mammography screening is?  What do you think could be done better? |
| Thank you for taking the time to tell us about your experiences!  Is there anything else you would like to add or mention that we have not discussed so far? |  |  |

**S3: Category system for experts**

| **(1) Challenges and practicability of the additional US** | | |
| --- | --- | --- |
| **Category** | **Abs. frequencies** | **Rel. frequency (%)** |
| Additional time required | 7 | 70 |
| Offer is not possible on the trailer | 3 | 30 |
| Additional staff required | 7 | 60 |
| Additional ressources (qualifications, devices) | 6 | 60 |
| False-positive findings, overdiagnosis | 6 | 60 |
| Quality of US examination is important | 8 | 80 |
| Technical problems | 1 | 10 |
|  |  |  |
| **(2) Reasons why women reject or accept the offer** | | |
| Scheduling problems | 6 | 60 |
| Language problems | 1 | 4 |
| No interest | 2 | 8 |
| Fear of additional examinations | 2 | 20 |
| Additional US is viewed positively | 5 | 50 |
| Women prefer US directly after mammography | 6 | 60 |
| Feeling of safety | 2 | 20 |
|  |  |  |
| **(3) Screening client’s knowledge of breast density in general and relevance of the topic for MSP** | | |
| There is no knowledge | 2 | 20 |
| Knowledge is heterogeneous | 1 | 10 |
| Mostly there is knowledge | 4 | 40 |
| The topic has not been neglected | 5 | 50 |
| The topic has been partially neglected | 2 | 20 |
| It is unproblematic to explain the topic to the clients | 1 | 10 |
|  |  |  |
| **(4) Experiences and beliefs regarding additonal US** | | |
| Addtional safety | 11 | 90 |
| No additional radiation exposure | 1 | 10 |
| US is not helpful | 2 | 20 |
| MSP could be considered ineffective by laypersons | 3 | 30 |
| US might only detect small and less aggressive tumours | 2 | 20 |
| Effect on interval cancers still unclear | 2 | 20 |
| US is time-consuming and visible effect is only small | 5 | 50 |
| Impact on overall breast cancer mortality unclear | 1 | 10 |
| Too much attention is paid to the problem of density | 1 | 10 |
|  |  |  |
| **(5) Relevance of the topic for the MSP** |  |  |
| Breast density is problematic for the MSP | 4 | 40 |
| Tomosynthesis as additional examination | 5 | 50 |
| Screening should not cause trouble | 2 | 20 |
| MSP alone is very effective | 2 | 20 |
| MSP is a mass examination | 3 | 30 |
| Doctors have a great responsibility | 1 | 10 |
| MSP has partly a poor image | 1 | 10 |
|  |  |  |

**S4: Category system for screening clients**

| **(1) Prior knowledge on breast density** | | |
| --- | --- | --- |
| **Category** | **Abs. frequencies** | **Rel. frequency (%)** |
| Heard of the topic for the first time | 9 | 40 |
| I already knew that I have dense breasts | 12 | 54 |
| I didn’t know I have dense breasts | 10 | 45 |
| Had an ultrasound before | 4 | 18 |
| Has impact on MX, understood study information | 20 | 90 |
| Dense breasts have no influence on diagnosing | 1 | 4 |
|  |  |  |
| **(2) Feelings about the diagnosis** | | |
| I felt neutral | 15 | 68 |
| It was not explained to me well | 2 | 9 |
| I was worried after the diagnosis | 3 | 13 |
| I would have liked more information about it | 3 | 13 |
| Everything was explained to me well | 5 | 22 |
|  |  |  |
| **(3) Experience with the additional examination** | | |
| Increased feeling of security | 13 | 59 |
| Examination is completely uncomplicated | 10 | 45 |
| I would like to have the additional US soon | 8 | 36 |
| Waiting time was ok | 9 | 40 |
| Like to have the US on the same day | 2 | 9 |
| I got the US on the same day, which was good | 6 | 27 |
| Waiting time was stressful | 2 | 9 |
| Quality of the examination depends on the device | 1 | 4 |
| US examination was very quick | 1 | 4 |
| I did not receive a debriefing | 1 | 4 |
| US examination was not pleasant | 1 | 4 |
| US should be performed by a woman | 1 | 4 |
| I needed a puncture | 1 | 4 |
|  |  |  |
| **(4) Has the attitude toward the MSP changed?** | | |
| Attitude towards MSP has remained the same | 12 | 54 |
| I feel valued because of the additional US | 4 | 18 |
| Do I need mammogram or is the US sufficient/better? | 3 | 13 |
| Positive, because no additional radiation exposure | 1 | 4 |
|  |  |  |
